# Supporting Oral Language Development in Preschool Children Through Instructional Scaffolding During Drawing Activity: A Qualitative Case Study

**DOI:** 10.3390/bs15070908

**Published:** 2025-07-04

**Authors:** Mengyun Xiao, Fadzilah Amzah, Noor Azlina Mohamed Khalid, Weihan Rong, Xiaolong Zhou

**Affiliations:** 1 School of Educational Studies, Universiti Sains Malaysia, Penang 11800, Malaysia; 2 School of the Arts, Universiti Sains Malaysia, Penang 11800, Malaysia; 3 College of Art and Design, Qufu Normal University, Rizhao 276826, China; 4 Faculty of Education, East China Normal University, Shanghai 200062, China

**Keywords:** preschool children, oral language development, instructional scaffolding, drawing activity, qualitative case study

## Abstract

The research on teaching scaffolding for preschool children’s oral language development (OLD) has become an important topic in the academic world. However, there remains a lack of evidence-based research on the integration of scaffolding strategies integrated into creative art contexts to support children’s creative expression and language production. In this study, a qualitative case study was conducted to analyze the non-participatory observation and artwork analysis of five-year-old children’s drawing activities in a kindergarten in China based on socio-cultural and scaffolding theories. Three types of core scaffolding strategies were summarized. The findings reveal that the three strategies work together dynamically within the children’s Zone of Proximal Development (ZPD): (1) the visual prompt strategy enriches the vocabulary diversity of metaphors, adjectives, and ordinal words; (2) dialogic narrative co-construction effectively improves narrative coherence across exposition, rising action, climax, and resolution; and (3) emotional engagement strategies foster a safe expressive environment, promoting the integration of affective vocabulary with intrinsic motivation. Accordingly, a three-dimensional integrated “visual-linguistic-emotional” scaffolding model was constructed, emphasizing the practical guidelines of simultaneous scaffolding and gradual scaffolding withdrawal during the warm-up, creation, and sharing sessions of the drawing activity. This study expands the application of scaffolding theory in unstructured art contexts, and provides a systematic practical framework for the design of cross-contextual language support strategies and teacher training in preschool education.

## 1. Introduction

Oral language development (OLD) in early childhood has been demonstrated to be fundamental to children’s cognitive growth, social competence, and later academic achievement (Jensen et al., 2015; West et al., 2024). Effective verbal communication skills enable children to better express their needs, share ideas, and participate in social interactions (Ramadan Elbaioumi Shaddad & Jember, 2024). In recent years, a growing body of research has focused on the effectiveness of teachers in promoting children’s language development through intentional scaffolding strategies, particularly in highly interactive teaching activities (Koyuncu et al., 2023; Voltmer et al., 2021). In this context, creative arts activities, and particularly drawing activities, provide a natural and engaging environment for children to express themselves (Ainsworth & Scheiter, 2021), as well as an important context for observing and supporting children’s language development. Despite the wealth of research in recent years demonstrating how drawing activity supports preschool children’s OLD (Hadley et al., 2022; Xiao et al., 2023a), studies that systematically focus on identifying which specific scaffolding strategies teachers employ during drawing activities, and how those strategies differentially influence children’s lexical diversity and narrative coherence, remain scarce. Indeed, the existing literature largely offers broad evaluations of overall scaffolding effects or relies on quantitative analyses in structured contexts (Ertugruloglu et al., 2023). By contrast, there remains a lack of systematic examination of the ways in which teachers and children interact in open-ended, semi-structured drawing tasks and which scaffolded behaviors are most targeted and actionable in these settings.

Therefore, this study focuses on teachers’ scaffolding practices during drawing activities to explore their role in supporting preschoolers’ OLD. The following section reviews the existing literature on preschoolers’ OLD, the role of instructional scaffolding in early education, the significance of drawing activities for language expression, and the theoretical foundations of this approach.

### 1.1. OLD in Preschool Children

The preschool years are a critical period for the rapid development of children’s oral language skills (Cabell et al., 2022). Lexical diversity and narrative coherence are widely recognized as two core dimensions in the development of expressive oral language (Z. Li et al., 2025; Pentimonti & Justice, 2010; Vettori et al., 2024). Vocabulary diversity refers to the richness of the various vocabularies used by children in natural communication or specific tasks, reflecting their semantic reserve and expressive flexibility (J. S. Yang et al., 2022). On the other hand, narrative coherence, refers to children’s ability to organize story units using chronological order, cause-and-effect logic, and a complete plot structure, and is an important reflection of the combination of language structure and thought organization (Otwinowska et al., 2020). Recent research continues to support this view; for instance, narrative coherence has been found to correlate with children’s mentalizing complexity in both fictive storytelling and autobiographical narratives (Foldager et al., 2024). In addition, studies on children’s comprehension of both linguistic and visual narratives provide further evidence that narrative coherence is closely tied to cognitive development and language processing (Adornetti et al., 2022). Moreover, it has been revealed that children’s development in these two areas in the preschool years is closely related to subsequent levels of reading comprehension, academic achievement, and social–emotional adjustment (Gillam et al., 2023; Khan et al., 2021; Z. Li et al., 2025). In interactive learning contexts, children’s verbal exchanges with adults and peers lead to the enrichment of vocabulary, increased sentence complexity, and the development of increasingly coherent and logical narrative skills (Washington-Nortey et al., 2022).

In preschool practice, teachers’ intentional creation of language-rich environments containing diverse interactive contexts can effectively support children’s improvement in both vocabulary use and narrative organization. Furthermore, it has been demonstrated that children’s language expression is more spontaneous during creative activities such as drawing and storytelling (Reese et al., 2023; Xiao et al., 2023a), and that supportive teacher interventions can further promote growth in vocabulary diversity and narrative coherence. Despite the large body of research focusing on children’s oral language in everyday communication and structured instructional activities, there remains a lack of systematic exploration of the mechanisms by which teachers support children’s vocabulary and narrative development through pedagogical scaffolding strategies in more open-ended, creative drawing activities. Thus, it is in this context that this study explores teachers’ practices of implementing scaffolding strategies in drawing activities to support preschoolers’ OLD.

### 1.2. Instructional Scaffolding in Early Childhood Education

First proposed by Wood et al. (1976), the concept of instructional scaffolding refers to the provision of timely support and guidance by adults during children’s learning process, according to their current level of competence, in order to help children accomplish tasks that they would not otherwise be able to complete independently. Scaffolding includes cognitive guidance, but also involves emotional support and social interaction to promote children’s independence, enabling them to learn on their own through progressively adjusted help (Vygotsky, 1978). Instructional scaffolding is widely used in preschool settings to support children in the areas of language development, and cognitive construction (Hadley et al., 2022; Skene et al., 2022). Teachers use strategies such as questioning, modeling, extending, and giving feedback to help children understand new concepts, organize language expressions, and improve communication skills in interaction. Effective scaffolding enhances children’s immediate performance while also promoting their motivation and long-term development (Koyuncu et al., 2023). Meanwhile, the ability of teachers to dynamically adjust scaffolding, i.e., to flexibly change the support approach according to children’s actual responses, has been identified as an important factor in enhancing scaffolding effectiveness (Van De Pol et al., 2010). Recent studies have reaffirmed the significance of contingent and adaptive scaffolding in current classroom practices (Muhonen et al., 2016; Pollarolo et al., 2024; Salminen et al., 2021), highlighting its role in promoting individualized learning and sustained engagement.

Furthermore, scaffolding also involves the facilitation of emotional and social interaction dimensions. Research has shown that teachers who give positive feedback and pay attention to children’s emotional states during scaffolding can enhance children’s self-confidence in expression and willingness to communicate verbally (Zhu & Lin, 2024). In addition, the application of scaffolding in social contexts, such as cooperative learning and group interaction, also helps to enhance children’s linguistic complexity and interactivity (Homayouni, 2022).

Although the positive effects of scaffolding in preschool education have been widely documented, most of the available research to date has focused on structured instructional activities or formal classroom conversations. Thus, the detailed process of the application of scaffolding in more open and naturalistic creative activities, its specific impact on children’s language development, remains to be further explored.

### 1.3. Drawing Activity as a Context for Language Development

Drawing activities are often seen as an important way to stimulate children’s creativity and expression (Saefurrohman, 2024; Wright, 2007). Through drawing, children present their ideas and emotions in visual form, with verbal communication naturally occurring in the process of description and sharing (Cox, 2005; Imafuku & Seto, 2022). Research has shown that children’s drawing activities are often accompanied by narratives of images involving object naming, plot construction, and the telling of personal experiences, and that this contextual language output contributes positively to children’s OLD (Ahn & Filipenko, 2007; Long et al., 2024). Consequently, Ainsworth and Scheiter (2021) recognize that drawing should be used extensively and proved its advantages in different ways.

Various scholars have emphasized the multiple properties of language development in the drawing context. On the one hand, drawing provides children with low-pressure, free expressive opportunities to use language spontaneously without the constraints of formal classroom structures, enhancing expressive initiative and narrative coherence (Zhang et al., 2024). On the other hand, research has also pointed out that in the absence of effective adult guidance, children’s language output may be limited to superficial descriptions, hindering the development of complex narratives or emotional depth (Heath & Wolf, 2005; Zakaria et al., 2021). Thus, the potential of drawing activities as a language-generating environment depends to a large extent on the ways in which educators intervene and the quality of their interactions.

Although drawing activities create rich opportunities for teachers to provide immediate language support in naturalistic contexts, existing research has mainly focused on the analysis of children’s autonomous narratives and drawing outputs (Costa et al., 2025; Podobnik et al., 2024). To date, there is still a lack of systematic exploration of the ways in which teachers specifically implement pedagogical scaffolding during drawing, and the mechanisms by which these scaffolding strategies contribute to children’s language organization and expression. Therefore, the practice of scaffolding in drawing activities is considered an important area that merits in-depth exploration.

### 1.4. Research Questions

The socio-cultural theory proposed by Vygotsky (1978) emphasizes that children’s linguistic and cognitive abilities are gradually constructed through social interaction and cultural practices. Thus, children are able to realize the process of transformation from external social experience to internal psychological functioning through collaboration and interaction with more experienced people. By interacting with more experienced adults or peers, children gain support within the Zone of Proximal Development (ZPD), which in turn drives the development of language competence (Cun & Cheng, 2023; Oliver & Azkarai, 2017; Zuckerman, 2007). In this context, teaching and learning activities are not simply a process of knowledge transfer, but an important arena for negotiating social meanings and practicing language. Furthermore, the instructional scaffolding theory further enriches the understanding of social interaction support mechanisms in socio-cultural theory. According to the theory, adults can provide dynamically adjusted support targeted towards children’s developmental levels as they complete tasks, thereby helping children to complete tasks independently and gradually removing scaffolding at appropriate time to promote autonomous development. Based on the above theoretical perspectives, this study focuses on drawing activities as a creative and contextualized educational practice, and explores how teachers can implement pedagogical scaffolding in this process to support the key dimensions of preschool children’s expressive speaking, for example, lexical variety and narrative coherence. The research questions for this study are as follows:

RQ1: How do teachers implement instructional scaffolding strategies during drawing activities in preschool classrooms?

RQ2: How do these scaffolding strategies support preschool children’s lexical diversity and narrative coherence in expressive oral language?

## 2. Materials and Methods

Due to the characteristics and practical considerations of this study, the qualitative case study method was used. This method is suitable for exploring the experiences and behaviors of participants in complex educational situations (Merriam, 2009; Priya, 2021). Furthermore, it allows for focusing on teachers’ support for the development of preschool children’s oral language skills through pedagogical scaffolding in teaching and learning interactions. Therefore, a purposive sampling strategy is appropriate to identify the study population based on the richness of information abundance, thus ensuring representativeness and depth of information (Ahmad & Wilkins, 2025; Patton, 2014). The triangulation of data sources, including non-participant observation and children’s drawings to obtain information, provided a solid foundation for understanding the relationship between scaffolding strategies and OLD. Notably, the data used for the study were selected from a portion of the first author’s doctoral project, which was conducted within the same kindergarten, and focused on two of the drawing activity segments for in-depth analysis.

### 2.1. Research Setting

A kindergarten affiliated with a public comprehensive university in Shandong Province, China, provided the setting for this research. Built in 1974, the kindergarten has continuously been recognized as a provincial model kindergarten by the Shandong Provincial Department of Education from 1998 to 2023, being well equipped with teachers and educational resources. The campus occupies an area of approximately 6000 square meters, with 10 teaching classes, 44 teaching staff, and over 300 children, covering three grades (K1, K2, K3).

The study was conducted with a class in grade K3. There were 30 five-year-old children in the class and the language of instruction was Mandarin. The kindergarten focused on the educational philosophy of integrating art and language; drawing activities were part of the teaching routine and were conducted regularly on a weekly basis. Mainly, they were conducted in formal art teaching classes and extended hours programs. Additionally, the kindergarten has a high level of language education input and practical experience in art teaching, which is highly representative and relevant and provides a suitably rich and authentic scene environment of language expression and scaffolding interactions for this study.

### 2.2. Participants

A purposive sampling strategy was utilized in this study to ensure that the selected participants would be able to provide rich, varied data on language interactions relevant to the topic of the study (Choi et al., 2023; Patton, 2014). Participants included six five-year-old children (three boys and three girls) and one female kindergarten teacher. Child participants were selected through purposive sampling. All children had been assessed at the end of the previous semester by their lead teacher using the Chinese version of the MacArthur–Bates Communicative Development Inventories (Chinese-CDI) (P. Li et al., 2007; Yue et al., 2021) to measure expressive language output. Raw scores were converted to a 1–5 standard scale in accordance with the normative manual. To ensure sufficient oral-language proficiency for the drawing tasks, only those scoring ≥ 3 (average or above) and demonstrating consistent verbal engagement in daily classroom activities, as confirmed by teacher observations, were included. The “Language skills level” column in Table 1 reflects each child’s Chinese-CDI expressive score (3 = average; 4–5 = above-average). This instrument was selected because it is a widely validated tool for assessing early expressive language skills in Mandarin-speaking preschoolers, and remains relevant in recent cross-linguistic developmental research (Marchman & Dale, 2023). Furthermore, child participants were recommended by the teacher to ensure that they could effectively interact verbally during the drawing activities and were appropriate for the study topic in terms of their cognitive and language developmental levels.

The children’s inclusion criteria referenced commonly used indicators for the language assessment of preschoolers (Justice et al., 2008; Q. Yang et al., 2024) and, in conjunction with the purpose of this study, specifically included (1) an average age of five years, (2) full participation in daily group instructional activities, and (3) teacher evaluations of oral language expression skills at an average or above-average level. Together, these selection criteria ensured that the interactive processes and linguistic outputs between instructional scaffolding strategies and children’s linguistic expressions could be effectively observed during the study. Table 1 presents the demographic information of the child participants. Gender and ethnicity were recorded to reflect sample diversity; however, these factors were not treated as analytical variables in the study.

With regard to the teacher, she had five years of experience in teaching young children, and her teaching style had been positively evaluated by both parents and school leaders. Furthermore, she specialized in drawing and frequently incorporated drawing activities into her daily teaching, demonstrating strong skills in integrating artistic and educational content.

It is important to note that this study was approved by the Ethics Committee of Universiti Sains Malaysia (protocol code USM/JEPeM/PP/24020183) as it involved an element of human data. Informed consent was provided by the participating teacher. Written informed consent was also obtained from the children’s guardians regarding the use of classroom observations and drawings. All participants were guaranteed of the anonymity of information and the confidentiality of the data.

### 2.3. Data Collection Instruments and Procedure

Data sources included non-participatory observations (video recordings, field notes) and children’s drawings. Multiple sources were used to achieve the triangulation of information in order to enhance the trustworthiness and richness of the study. The study focused on teachers’ implementation of instructional scaffolding during drawing activities and their role in supporting children’s OLD. All classroom interactions were conducted in Mandarin, after which the video and text data were translated into English by the research team, with multiple rounds of proofreading to ensure semantic accuracy and cultural equivalence.

The drawing activity was based on the theme of “twelve Chinese zodiac signs”, which covers a variety of animal figures. In this paper, the focus is on the theme of the rabbit, which was chosen for the following reasons: (1) It is familiar to children and has cultural associations. The rabbit is one of the most common images in traditional Chinese culture (Shen et al., 2023). It appears frequently in picture books, animation, and nursery rhymes, which is in line with preschoolers’ life experience and cultural background, and is conducive to activating their association and expression. (2) It is a age-appropriate image and easy to depict figuratively. As a gentle and well-liked animal, the rabbit has a simple and maneuverable image, which is suitable for the drawing development level of five-year-old children. (3) It stimulates interest and emotional projection. Pre-interviews and teacher observations demonstrated that children generally held positive emotions toward the rabbit image, which could stimulate their motivation to participate. (4) It offered full and sufficient data. The two drawing activities included in this theme provided complete teaching scaffolds and language performance fragments, which are representative of the depth of analysis. Therefore, this theme was selected as the sample material for in-depth analysis.

For each theme, the researchers and teacher collaborated to design two drawing activities. Each activity was based on a scenario provided by the teacher, with the children being guided to draw freely based on the scenario. Before the activities, the teacher was introduced to the overall three-phase structure of the session: warm-up; drawing and scaffolding; and sharing and briefing on the general objectives of the study. However, no specific scripts or prescriptive scaffolding instructions were provided; rather, the teacher was encouraged to apply her professional judgment and interact freely with the children while implementing the drawing sessions. The two specific scenarios analyzed in this study were “Rabbit Goes to a Party” and “Rabbit Participates in Sports Events”. Both drawing activities were organized in two consecutive weeks of regular class time; each activity lasted approximately 35 min, and was structured in three sessions, as shown in Table 2.

**Figure 1:**
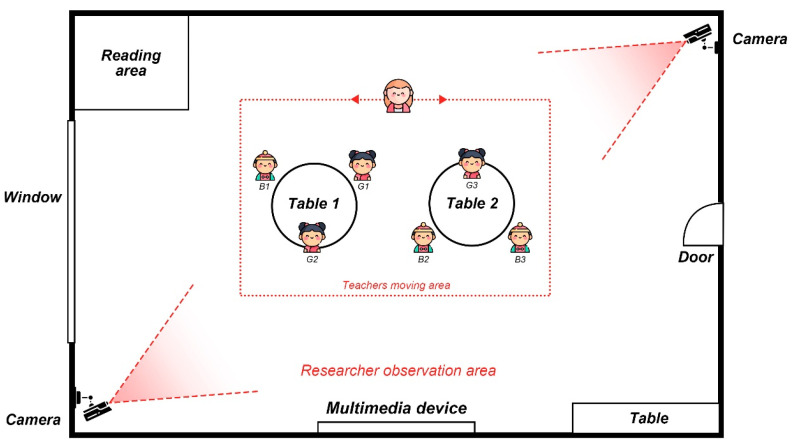
Layout of classroom observation (source: authors’ illustration).

A total of 70 min of classroom video footage was collected for the study, which was recorded in its entirety by a fixed camera (see Figure 1). Simultaneous field notes were written to capture interactive information, such as the teacher scaffolding language and the children’s verbal responses. All activities were conducted in Mandarin, after which the audio visual recordings were transcribed and translated into English by the research team, with multiple rounds of proofreading to ensure the cultural and semantic equivalence of the translations.

In addition, the study collected 12 drawings from six children as an important supportive material for the observation analysis (Appendix A). These works corroborated the observation records, providing multidimensional evidence for understanding the link between teacher scaffolding behaviors and children’s oral language performance (Yin, 2014). The multimodal data collection strategy used in this study was able to reveal more systematically the development of vocabulary diversity and narrative coherence in preschool children supported by specific scaffolding strategies in the drawing context.

### 2.4. Data Analysis

Thematic analysis allows for the systematic categorization and interpretation of observational data and children’s drawings (Braun & Clarke, 2006; Kiger & Varpio, 2020). Thus, it is suitable for exploring the implicit construction of meaning in complex educational interactions and can help to reveal the relationship between teachers’ scaffolding behaviors and children’s language. In addition, to improve the systematicity and transparency of the analysis, the research team used NVivo 14 software for data organization and coding. First, the researcher transcribed the text of the video data recorded during the drawing activity in order to record the content of the teacher–child language interactions. Simultaneous crosschecking was performed with the classroom observation field notes to ensure that the information was complete and semantically accurate. As the research context was in Mandarin Chinese, the transcriptions were translated into English after review by the research team to ensure terminological consistency and cultural equivalence. Next, the researchers imported the transcribed text into NVivo. Nodes were used to manage the coded information, and combined word frequency analysis, language fragment comparison, and image content assistance were applied to initially identify linguistic behavioral features related to the research questions. The study used a combination of open coding and theme induction, whereby the data were first analyzed and coded line by line before similar codes were continuously aggregated to summarize the themes underlying the research question (Braun & Clarke, 2006).

Additionally, it is important to mention the strategy that the researcher employed in order to enhance the credibility and interpretive validity of the study. Independent coding and initial thematic summarization were conducted by each of the two authors, followed by multiple rounds of negotiation and discussion to reach a consensus on coding in the case of discrepancies. Any unclear themes were redefined or merged until, eventually, three themes were identified as best reflecting the relationship between teachers’ scaffolding practices and children’s language development. Representative discourse passages and drawings were selected from them as supporting materials. Importantly, to enhance research ethics and ensure data protection, all the corpus involved was deidentified, with participant identities being replaced with codes to ensure that their privacy remained intact (Dougherty, 2021; Guest et al., 2013).

### 2.5. Trustworthiness

To ensure the credibility of this study, a combination of strategies were utilized during its implementation, such as member checking, peer review, triangulation, and thick description (Merriam, 2009; Priya, 2021). During the data analysis stage, the researchers fed back the initial findings to the teachers, gaining confirmation of the accuracy of the interview results and interpretations. Multiple rounds of review and discussion followed with two experts in the field of education (child language development and arts education) in order to enhance the soundness of the research interpretations. Furthermore, to increase the trustworthiness of the data, multiple sources of data, including non-participant observation (video footage and field notes), teacher interviews, and children‘s drawings—were used for cross-validation. In addition, the study enhanced the comprehensibility and applied transfer value of the findings by providing thick descriptions of the research context, participant characteristics, and teaching scenarios. Collectively, all of the abovementioned strategies help to cement the foundation of credibility underpinning this study, conferring high explanatory power as well as practical educational significance on the findings of the study.

## 3. Results

The aim of this study was to analyze the ways in which teachers use pedagogical scaffolding to support the development of preschool children’s oral language skills during drawing activities. Three themes were identified from the data analysis: language elicitation through visual prompts; the co-construction of narrative through dialogic interaction; and emotional engagement within creative contexts. Two subthemes were further refined under each theme, and the structure of the themes and subthemes summarized in this study is depicted in Table 3. The following subsections describe and analyze each theme in relation to the observation segments and children’s work.

### 3.1. Theme A: Language Elicitation Through Visual Prompts

In drawing activities, the teacher points to image details and asks open-ended questions to guide children to observe and verbalize their thoughts. Together, the scaffolding strategies of Visual Focus Guidance and Linguistic Prompting constitute an important driving mechanism for children’s vocabulary diversity development. Visual Focus Guidance refers to prompting children to observe and describe by pointing to key elements in the picture (R. Li et al., 2024; Otto, 2006). Linguistic Prompting describes the use of questions or exclamations to elicit more specific descriptions (Pentimonti & Justice, 2010; Wu et al., 2025). In practice, the combination of these two approaches inspires children to go beyond simple naming and use more varied and metaphorical language. In this subsection, two concrete examples of drawings were used to gradually guide children to develop language extensions such as metaphorical expressions, adjective stacking, and ordinal constructions. Table 4 depicts the ways in which children’s language changed from primitive to post-developmental when scaffolded in the C4 and C5 modalities.

**Figure 2:**
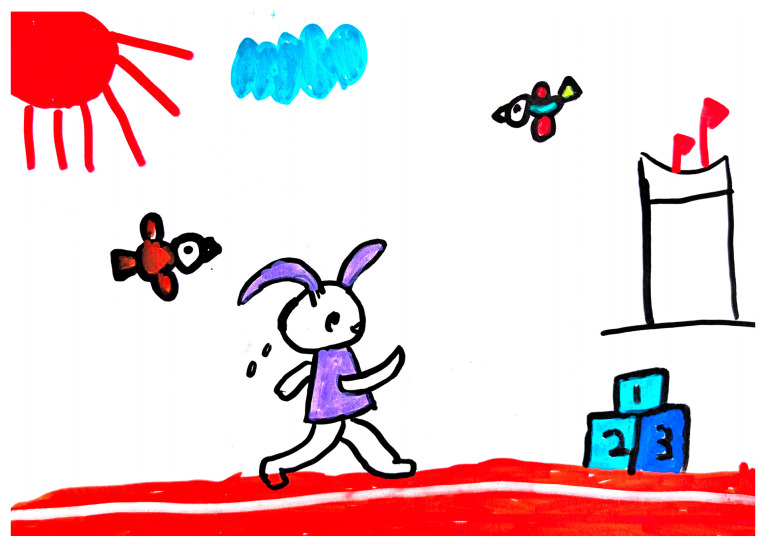
C4’s “Rabbit Participates in Sports Events” drawing (source: child’s drawing).

For “Rabbit Participates in Sports Events”, C4 created a drawing depicting a rabbit running on a red track. The picture also included birds watching the race, a high podium, and the sun (see Figure 2). By pointing to the “running rabbit,” the teacher asked, “Is it fast? What does it look like?” C4 initially responded, “It’s running.” The teacher then adopted a follow-up strategy that successfully elicited C4’s use of metaphorical language such as “like the wind”. This linguistic output demonstrated children’s scaffolded transition from behavioral naming to figurative analogies, and the use of similes demonstrated the activation of semantic abstraction. After the teacher praised the children, C4’s active and metaphorical language increased significantly. Later in the same activity, when the teacher continued to direct the children to look at their work (pointing to the flying bird and the podium), the children responded with ordinal words, reflecting their use of ordinal adjectives and event lexical combinations, and demonstrating an increase in lexical refinement in context (see Table 5 for details).

**Figure 3:**
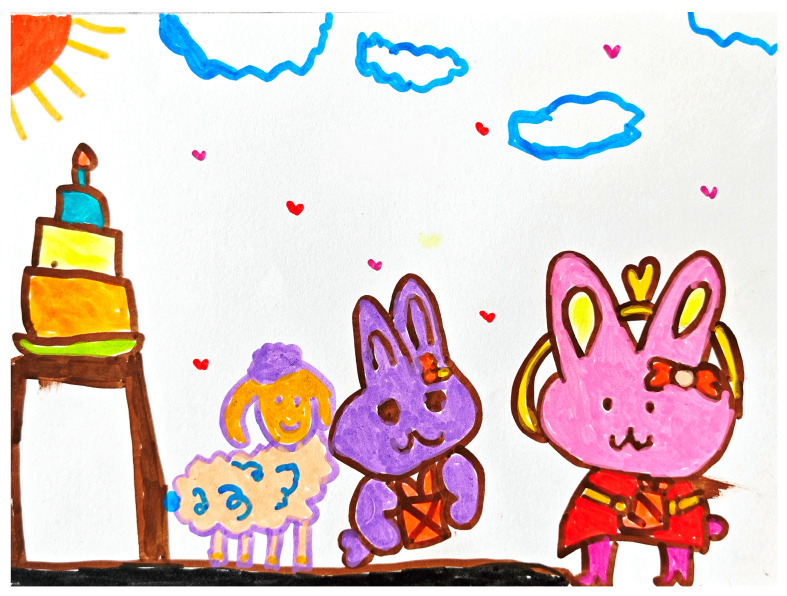
C5’s “Rabbit goes to a Party” drawing (source: child’s drawing).

In another activity context, “Rabbit goes to a Party”, the scaffolding strategy also demonstrated a positive effect on children’s oral production, as C5’s drawing (see Figure 3) depicted the character of a rabbit standing next to a cake, dressed up and smiling. Through visual guidance and Linguistic Prompting, the teacher continuously stimulated C5’s observation and expression of the details of the picture, which facilitated the expansion of oral language from initial vocabulary to a variety of expressions.

Early in the activity (after approximately six minutes), the teacher posed an open-ended question, “What’s your rabbit doing? Where is it going?” to which C5 initially responded succinctly. However, during subsequent compositions, as the images became richer in content, the teacher continued to guide children to use more descriptive vocabulary by pointing to details of the image (e.g., the rabbit’s attire, the cake decorations, etc.) to open up the question. After approximately fifteen minutes, C5 was able to independently use color words, adjectives, and complex sentences such as “red dress, golden bow,” and “three-layer cake for the party”. Thus, this process of language production clearly demonstrates the positive role of the teacher in scaffolding to support children’s vocabulary diversity, as shown in Table 6.

Table 7 shows only the number and level of occurrences of each type of novel word in the warm-up, scaffolding, and sharing phases for the two typical participants to show the differential impact of scaffolding on vocabulary diversity. In both typical cases, the frequency of similes and adjectives in the scaffolding session rose to a moderate level, suggesting that the teacher’s visual focus and verbal cues successfully prompted the children to describe the picture in more vivid language. In the sharing session, the frequency of emotion words climbed further, reflecting both peer and teacher encouragement to stimulate stronger emotional expression in oral language.

Together, these examples indicate that teacher scaffolding, even within short exchanges, enabled richer, more diverse vocabulary use among preschoolers, advancing OLD. Furthermore, the findings underscore that strategically timed prompts can rapidly expand children’s expressive capacity and that scaffolded techniques are effective in supporting language growth within creative activity contexts.

### 3.2. Theme B: Narrative Co-Construction Through Dialogic Interaction

Theme B of this study consists of two subthemes: Subtheme B-1, Open-Ended Questioning, and Subtheme B-2, Narrative Extension Scaffolding. The former involves stimulating children’s initial construction of plot beginnings and character motivation. The latter supports children to extend story details and form structured narratives within the framework of “exposition, rising action, climax, resolution” by asking questions relating to process, outcome, and emotion. In order to emphasize the typicality, Case C3 from “Rabbit Participates in Sports Events” and Case C1 from “Rabbit Goes to a Party” were selected for in-depth analysis. These cases were selected because they demonstrated narrative trajectories and interaction patterns that were commonly observed among the participants. Both children received average to high scores in narrative coherence, and their dialogs reflected recurring scaffolding moments shared by other cases. Although individual variations existed, these two examples represented typical responses in terms of teacher-child interaction structure and child language performance under scaffolding.

**Figure 4:**
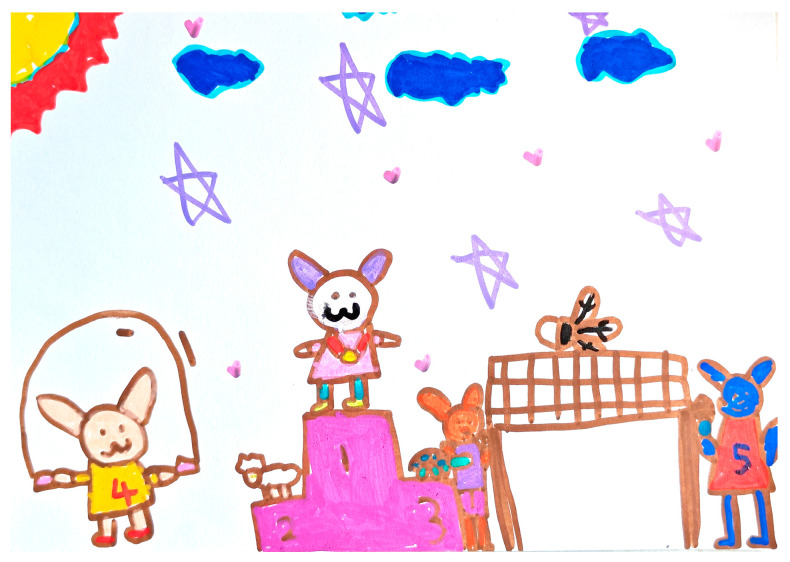
C3’s “Rabbit Participates in Sports Events” drawing (source: child’s drawing).

In the “Rabbit Participates in Sports Events” drawing activity, child C3 depicted multiple characters participating in a competitive event, with rich elements such as the high jump, rope skipping, a tennis match, a podium, and audiences (as shown in Figure 4).

Under the guidance of the teacher, C3 named and explained the image elements. Gradually, C3 also constructed a complete background and developmental sequence of events, showing a preliminary ability to start and finish a narrative. The dialog segment in Table 8 presents C3’s narrative generation process during the activity of drawing, reflecting the teacher’s process of “narrative co-construction”. Children were guided to integrate scattered images into a logical and coherent oral language narrative. In this case, C3’s initial naming of a single element in the scaffolding session (“tennis net”) gradually transitioned into a full causal narrative (“it jumped the highest” to “won the championship” to “celebrated with the audience”). Narrative coherence increased from fragmentary descriptions to structured narratives, validating the effectiveness of the teacher’s dialogic scaffolding.

**Figure 5:**
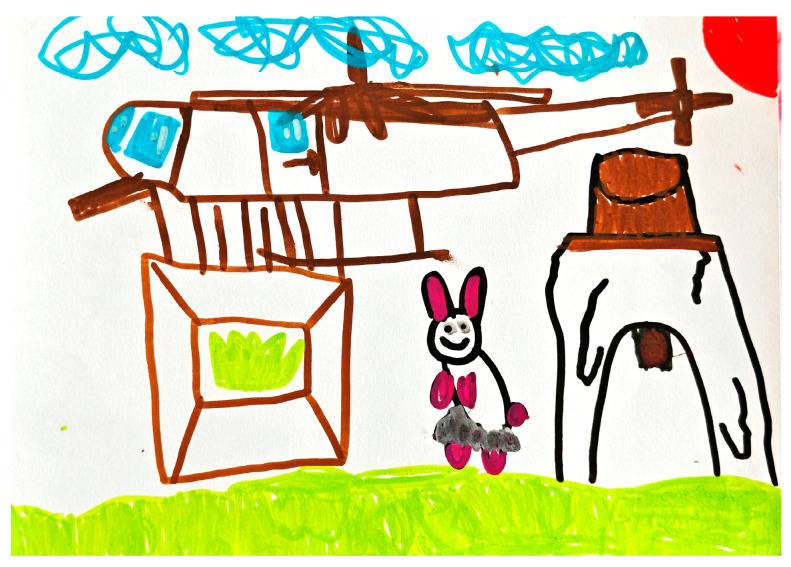
C1’s “Rabbit goes to a Party” drawing (source: child’s drawing).

In addition to the narrative power of C3 in “Rabbit Participates in Sports Events”, C1’s drawings and oral narratives in “Rabbit Goes to a Party” also fully demonstrate the support of dialogic scaffolding for narrative logic and event structure (see Figure 5). In fact, C1’s work transcends the conventional party scene and is highly fantastical and spatially imaginative. In the picture, the rabbit flies over the forest in a helicopter, aiming for a forest party. Through a series of open-ended questions and plot extensions in the teacher–child interaction, the teacher guided C1, step by step, in building a complete narrative example that included setting, conflict generation, and resolution, thus enhancing her narrative coherence and plot organization.

Table 9 shows the dialogic fragments of C1’s drawing process at three key points in time. It can be seen that the teacher’s dialogic scaffolding played multiple roles in narrative structure, role construction, and emotional experience. Guided by the teacher, C1 transitioned from initial single sentence responses (“to go to the party”, “to paint a helicopter”) to full narratives with cause-and-effect sequences (“it was going to dance and eat the carrot cake”), role relationships (“to go to the party with my friends”), and affective overtones (“it was very happy”). Such a developmental trajectory validates the synergistic effects of Open-Ended Questioning and Narrative Extension Scaffolding on children’s narrative coherence and imagination.

Having analyzed the cases of C3 and C1, Table 10 shows the overall impact of the group level on children’s narrative coherence in the narrative co-construction through dialogic interaction strategy for all participants. Using a scale from one to three, the researcher assessed the verbal narratives of the child participants in the drawing sharing session. As can be seen from the data in the table, the majority of children in Subtheme B-1 achieved moderate coherence, with only C5 and C6 showing low coherence. This indicates that the session was generally able to stimulate children’s construction of the starting point of the plot. Furthermore, in Subtheme B-2, the average group score was 2.5 (between moderate and high coherence). This suggests that the teacher was effective in facilitating children’s complete descriptions of the course of events by pursuing outcome, cause and effect, and emotion. Thus, the children’s developmental leap from image description to a complete story line further confirms the importance of the dialogic narrative co-construction strategy in enhancing children’s narrative coherence.

### 3.3. Theme C: Emotional Engagement Within Creative Contexts

During the creative drawing activities, the teacher focuses on children’s verbal output. Through emotional scaffolding behaviors such as intonation, encouraging comments, and empathic questioning, they create a safe and accepting environment for children to express themselves (Heath & Wolf, 2005). Centered on emotional resonance, this kind of scaffolding, centered on emotional resonance, enhances children’s confidence in expression, and encourages them to add more emotional color and psychological motivation to their language production process, thus achieving a double leap in both vocabulary richness and narrative coherence. In the following section, Subtheme C-1: Affective Scaffolding and Subtheme C-2: Empathic Interactional Support will be presented in conjunction with the sharing case of C2 and the drawing process of C6.

**Figure 6:**
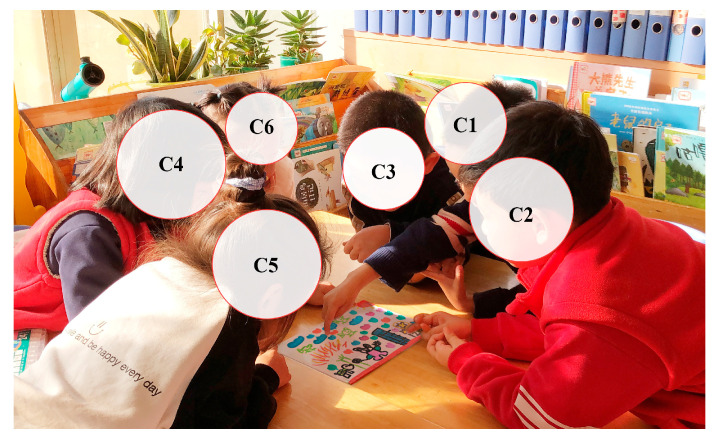
Peer engagement and emotional support during C2’s sharing moment (source: researchers’ photos).

During the sharing session, C2 was invited by the teacher to focus on and present their work to with their peers (see Figure 6). The following excerpt was recorded in the researcher’s field notes:

*C2 Pick up the drawing paper and whisper, “Here’s my drawing of the track, and the rabbit just won the race.” As the teacher approaches and smiles, say, “Awesome! Can you tell everyone what it’s thinking about now?” C2’s eyes light up and then add, “It wants to go home and tell its mom and have carrot cake to celebrate!” (FN, C2, Week 2, 21:40 min)*

Following this, C2 then used emotive words such as “celebration, cake, jumping, fireworks” in the description. Table 11 presents related dialogs. In this series of dialogs, C2’s initial single sentence “It won the race” was limited to a simple statement of the facts of the picture, which represents a basic description of the “exposition” stage of the plot. After the teacher repeatedly used encouraging questions and affective feedback, C2 expanded the sentence length and spontaneously introduced more affective verbs (“celebrated, jumped”) and specific nouns (“carrot cake, fireworks”). By organically combining the details of the image, emotional experience and action intention, the four-stage narrative structure of “Exposition, Rising Action, Climax, Resolution” is completed. During this process, the lexical diversity of C2 rapidly shifted from low-frequency generic nouns to medium–high-frequency emotion words and object nouns, while the narrative coherence also increased, proving that the two subthemes were more coherent in terms of emotional experience and action intention. Therefore, the central role of the two subthemes in the coupling mechanism of emotion-driven expression and speech production was demonstrated.

In addition, the researcher’s field notes were recorded during the non-participant observation of C6 during the middle of the Rabbit Goes to a Party event (approximately 14:20):

*C6 is coloring a gift box and repeating “gift for a friend” softly, with joy in her voice. Teacher A smiles and responds, “That’s great, she’s going to love it!” C6’s eyes light up and she draws a purple bow on the box. During the sharing session, C6 states to self, “She will jump up and down with joy to see the gift!” (FN, C6, Week 3)*

Table 12 presents the two critical affective guides given by the teacher to C6.

During this process, C6’s language rapidly shifted from short sentences to multi-sentence descriptions of the characters’ emotions and actions; additionally, emotional verbs and onomatopoeic expressions appeared in her vocabulary, reflecting a high degree of integration of intrinsic motivation and emotional coloring. Both cases demonstrate that Affective Scaffolding and Empathic Interactional Support strategies significantly enhance children’s use of emotional vocabulary and narrative coherence during the drawing creation and sharing sessions. Thus, this study demonstrates that the Affective Scaffolding strategy provides preschoolers with a safe space for expression and plays a key role in facilitating vocabulary enrichment and story structure generation.

## 4. Discussions

By conducting a qualitative case study, this study explored the process of imple-menting pedagogical scaffolds and examined their contribution to preschoolers’ vocabulary diversity and narrative coherence. In this section, the two research questions are developed and discussed, concluding with an integrative reflection.

### 4.1. Implementation Process of Instructional Scaffolding Strategies (RQ1)

In this study, the teacher used three scaffolding strategies, Visual Focus Guidance, dialogic narrative co-construction, and emotional engagement, to produce dynamic and progressive support effects at different points in the children’s drawing activities. Specifically, in the warm-up phase, the teacher employed visual focus to activate the children’s background experiences by pointing to key elements in the drawing scene and asking open-ended questions to trigger initial naming (Khaqan et al., 2025; Rivera et al., 2005). In the scaffolding stage, the teacher combined open-ended questions with other follow-up questions to extend the children’s verbal descriptions of the details in the drawings, thereby implementing a process of constant scaffolding and fading that helped the children to internalize external cues into autonomous expressive strategies (Salminen et al., 2021; Van De Pol et al., 2010). This finding supports Ainsworth and Scheiter’s (2021) argument that co-constructed understanding is more likely to be internalized during interaction. It is worth emphasizing that adults and children work side-by-side in natural creative activities, with the teacher both teaching what to say and inspiring how and why to say it. During the sharing feedback phase, the teacher created an atmosphere of safe ac-acceptance through encouraging comments and empathic responses, allowing the children to become bolder in their use of emotional vocabulary and complex sentences in their narratives, thereby accomplishing the final removal of scaffolding and transfer of responsibility (Deshmukh et al., 2022; Rogoff, 2003). Meanwhile, research on preschoolers’ motivational scaffolding strategies emphasizes that combining scaffolding with children’s pre-existing experiences and interests can further stimulate exploratory motivation (Ollonen & Kangas, 2025). Thus, the principles of scaffolding flexibility and child-centeredness highlighted by this finding illustrate the importance of dynamically adapting scaffolding to children’s developmental levels and situational needs.

### 4.2. Scaffolding Strategies for Lexical Diversity and Narrative Coherence (RQ2)

At the vocabulary level, the visual focusing and verbal triggering strategies significantly stimulated children’s use of novel word categories such as metaphors, adjectives and ordinal numbers, and nouns. For example, C4’s vocabulary richness increased from 0 to 2 for metaphors and from 1 to 2 for adjectives in the scaffolding session (see Table 4), marking a significant increase in vocabulary richness compared to pure naming. This suggests that in an open and creative drawing context, such prompts are more likely to activate children’s semantic association network, allowing them to move beyond describing the surface attributes of things and creatively use vivid expressions such as “like the wind” and “golden bow”.

In terms of narrative coherence, the teacher’s open-ended questioning and narrative extension scaffolding effectively supported the children in building a complete structure of “exposition, rising action, climax, resolution” (Xiao et al., 2023b). Mean group ratings showed that most children achieved moderate coherence in the co-construction session and high coherence in the sharing session, demonstrating the scaffolding strategy’s facilitation of causal and chronological organization. Once again, the teacher used open-ended questions to stimulate children’s exploration of character motivation, before guiding them to add process and outcome through refined follow-up questions, thereby enabling the children to achieve a complete narrative structure. This finding is consistent with Gardner-Neblett et al.’s (2024) study of constructive interaction as a means to promote children’s narrative skills, but this case further illustrates that the combination of open-ended questions and narrative extension scaffolding can be more effective in a drawing activity than in a traditional classroom in terms of evoking children’s sensitivity to cause and effect and chronology, resulting in a significant increase in narrative coherence, even in a short period of time. In addition, the scaffolding strategy further demonstrates the advantages of crossmodal support in art contexts, helping children to establish a strong connection between “drawing” and “speaking” and enabling them to make the transition from static images to dynamic narratives.

### 4.3. Integrative Modeling and Theoretical Reflection on Scaffolding

**Figure 7:**
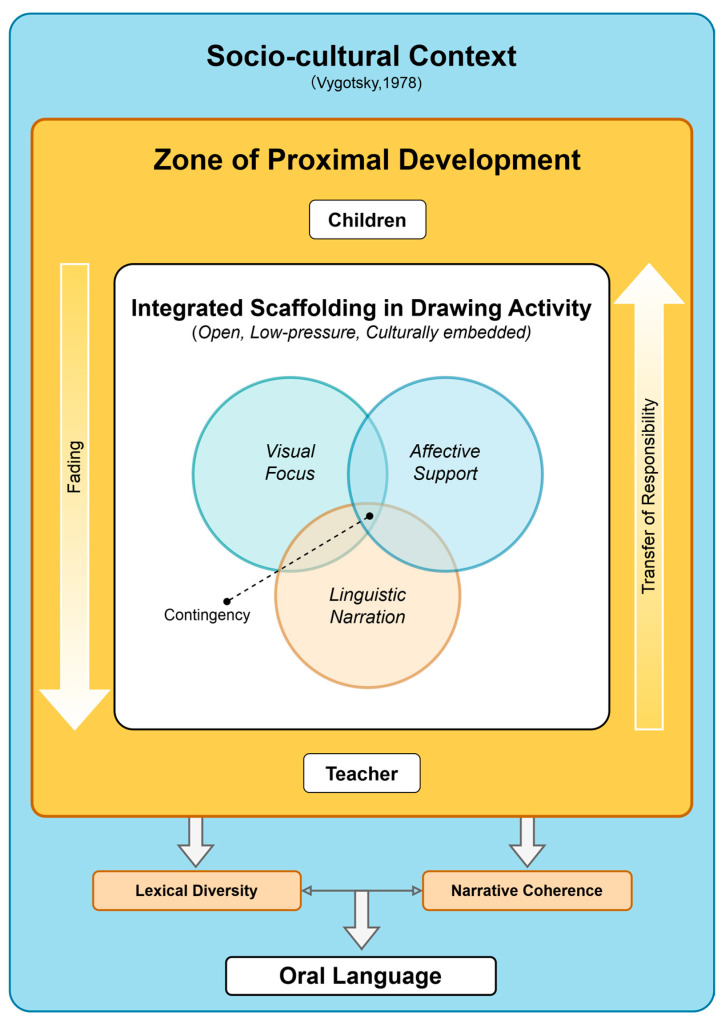
Three-dimensional integrated instructional scaffold framework (Vygotsky, 1978) (source: authors).

Based on the above findings, this study proposes a three-dimensional integrated scaffolding model of “visual, verbal, and emotional”, as shown in Figure 7. Following the macro-framework of Vygotsky’s socio-cultural theory (Vygotsky, 1978), the model views drawing activities as open and culturally embedded low-pressure contexts. Our findings emphasize the need for teachers to invoke the three types of scaffolding simultaneously within the child’s ZPD, transitioning from external cues to internalized expression through dynamic adjustment and gradual withdrawal paths. Theoretically, this study expands the applicability of scaffolding theory (Chen & Badolato, 2025; Wood et al., 1976) in unstructured art contexts and enriches the perspective of research on children’s OLD by incorporating emotional connections into the scaffolding framework of language production for the first time, thereby providing a novel contribution to the literature. In practical terms, the model offers preschool teachers operational guidelines for designing language support strategies in creative activities such as drawing and crafts, and helps to facilitate teachers in crafting immediate feedback and interaction, thus promoting the more effective synergistic development of children’s lexical diversity and narrative coherence in daily teaching.

Furthermore, it is worth emphasizing that, although the children came from different ethnic and gender backgrounds, the purpose of collecting demographic data was to reflect on the diversity of the sample, rather than to examine group-based differences. All of the children participated in the same Mandarin-based kindergarten program and received comparable instructional input. Therefore, neither ethnicity nor gender were treated as an analytical variable in this study, and no patterns related to these demographic factors were identified in the scaffolding interactions or in children’s oral language performance.

### 4.4. Limitations

Although the study represents a positive contribution of scaffolding strategies to lexical diversity and narrative coherence, there are nevertheless several limitations that need to be noted and mitigated in future research. First, while the main drawing activities in this study took place over two consecutive weeks, the overall data collection period spanned approximately two months. This included preparatory meetings with the teacher and classroom observations. As language development is highly cumulative and stage-specific, extending the observation period and tracking multiple teaching interactions in a similar open-ended art activity context would help to reveal the long-term effects of scaffolding strategies in children’s language development, especially in terms of the evolution of dimensions such as lexical depth and grammatical complexity. Second, this study adopted a qualitative case study approach with a small sample of six five-year-old children. Although data triangulation was employed, the limited sample size restricts the generalizability of the findings to broader educational contexts. Furthermore, while purposive sampling ensured active verbal participation, it may also limit the applicability of the results to children with different levels of oral language skills.

### 4.5. Future Research Directions

Future studies may expand the sample size or incorporate quantitative tools to enhance the explanatory power and generalizability of the study. In addition, although this study focused on language production in drawing activities, language development may also be activated and supported in other types of artistic expression, such as clay modeling, crafting, and dramatic play. In the future, it would be interesting for researchers to compare differences in the characteristics of teachers’ scaffolding behaviors within different artistic media sessions in order to explore their specific effects on the various dimensions of children’s language.

## 5. Conclusions

This study focused on instructional scaffolding practices in the context of drawing activities in a Chinese kindergarten, aiming to explore their role in supporting children’s vocabulary diversity and narrative coherence. The findings suggest that visual and verbal scaffolding effectively stimulates children’s observation and description of picture details, resulting in a significant enrichment of verbs, adjectives, and metaphorical expressions, as well as enhanced vocabulary diversity. Meanwhile, the study used a dialogic narrative co-construction strategy, in which the teacher guided the children to build up the sequence of events through open-ended questions and plot extensions, thus enhancing narrative coherence and logical organization. Furthermore, the study found that emotional scaffolding, centered on encouraging feedback and empathic interactions, creates a safe and accepting language environment, stimulating children to combine their inner emotions with language production, while enriching their levels of expression.

Three main innovations of this study can be seen at the theoretical level. (1) It breaks through the limitations of scaffolding theory, which has been traditionally applied to structured classroom dialogs, and successfully introduces Vygotsky’s socio-cultural and pedagogical scaffolding theories into an unstructured early childhood creative drawing context, empirically revealing that the operation mechanism of pedagogical scaffolding in the natural art activities is both complementary to and dynamically synergistic with the scaffolding theory. (2) This study is the first to systematically incorporate the dimension of emotional resonance into scaffolding research, highlighting that empathic feedback not only enhances children’s linguistic confidence, but also improves children’s narrative coherence. It also injects intrinsic motivation and emotional coloring into children’s narrative coherence, adding an important perspective to the traditional scaffolding framework that focuses on cognition and language. (3) The integrated scaffolding model proposed for young children’s drawing activities clarifies the path of scaffolding intervention, dynamic adjustment, and gradual removal in children’s ZPD. Furthermore, it enriches the understanding of “when to engage and withdraw” scaffolding in drawing, and provides a replicable framework for cross-cultural and multi-art form research.

At the practical level, the results of this research can significantly contribute in three key ways. (1) Based on the research findings, a three-phase scaffolding operational guide is proposed for young children’s drawing activities, described as follows. In the warm-up phase, visual cues are used to activate children’s background experience and open-ended questions employed to quickly assess their ZPD starting point. In the creation and scaffolding phase, visual focus, sequential questioning, and emotional response are synchronized with the core creation process to build vocabulary and narrative skills step-by-step, with support decreasing based on children’s performance. In the sharing phase, children are guided through peer–teacher interactions to consolidate new vocabulary and story structures, with the teacher providing immediate encouraging feedback to reinforce children’s willingness to express themselves. (2) This operationalized framework can be directly translated into teacher training modules to help teachers identify children’s language development levels in a timely manner; additionally, it will enable teachers to flexibly design multi-scaffolding discourse, and master the timing of scaffold removal. (3) Curriculum designers can also use this framework to preset language production goals and evaluation criteria (e.g., vocabulary diversity checklist, narrative coherence scale) in regular art, thematic activities, and cross-curricular programs to systematically improve the quality of art-lingual integration in preschool education.

## Figures and Tables

**Table 1:** Demographics of child participants.

Child Name	C1	C2	C3	C4	C5	C6
Gender	Male	Male	Male	Female	Female	Female
Ethnicity	Chaoxian nationality	Han Chinese	Han Chinese	Manchu	Hui nationality	Han Chinese
Age in months	59	61	62	58	63	61
Language skills level	Average	Average	Above-average	Average	Average	Above-average

**Table 2:** Session design in drawing activity.

Items	Content
Materials	(1) 1 paper per person (A5, 148 mm × 210 mm). (2) Colorful acrylic markers. (3) Pictures of rabbits.
Group	(1) 2 groups of 3 children each (as shown in Figure 1).
Structure	(1) Warm-up phase (5 min): The teacher activated children’s background knowledge and introduced the “Rabbit” theme and drawing scenario. (2) Drawing and scaffolding phase (25 min): Children engaged in free drawing while the teacher provided dynamic scaffolding strategies to encourage talking during the creative process. (3) Sharing phase (5 min): Children voluntarily shared their drawings with peers.

**Table 3:** Themes and subthemes.

Themes	Subtheme
Theme A: Language Elicitation through Visual Prompts	A-1: Visual Focus Guidance A-2: Linguistic Prompting
Theme B: Narrative Co-Construction through Dialogic Interaction	B-1: Open-Ended Questioning B-2: Narrative Extension Scaffolding
Theme C: Emotional Engagement within Creative Contexts	C-1: Affective Scaffolding C-2: Empathic Interactional Support

**Table 4:** Child language from “original” to “developed” under different scaffolding strategies.

Child	Scaffolding Strategy	Original Utterance	Developed Utterance	Change Features
C4	Analogous Questioning & Visual Focus Guidance	“It’s running.”	“It’s running like the wind!”	Added metaphor; introduced competition meaning.
C5	Detailed Observation Guidance	“Skirt.”	“Red skirt with a golden bow.”	Adjective piling; refined material/color detail.

**Table 5:** Dialogs and scaffolding in visual-language elicitation (C4).

Time	Talking Episodes	Teacher Scaffolding
00:08:15	Teacher A: “What is this rabbit you’re drawing doing? Where is it running?” C4: “It’s running.” Teacher A: “Is it running fast? Look what’s flying next to it.” C4: “Yes, like the wind. That’s little birds, flying in the sky, they are watching the race.” Teacher A: “Wow, like the wind, what an awesome rabbit!”	Subtheme A-2: With “Is it running fast?” “What does it look like?” and other questions to promote rich expression.
00:21:42	Teacher A: “And what’s this one? (points to podium)” C4: “That’s where the prizes are won, first place, second place, and third place.”	Subtheme A-1: Point to the flying bird and the podium for guided observation.

Notes: In the above dialog segment, C4 produced 2 similes and 1 ordinal word in the scaffolding phase.

**Table 6:** Dialogs and scaffolding in visual-language elicitation (C5).

Time	Talking Episodes	Teacher Scaffolding
00:06:15	Teacher A: “What’s your rabbit doing? Where is it going?” C4: “It’s running.”	Subtheme A-2: Eliciting initial verbal responses through non-leading questions.
00:15:35	Teacher A: “Who is this rabbit you’ve drawn?” C5: “She’s the Rabbit Queen.” Teacher A: “What is she wearing?” C5: “A red dress and a golden bow.” Teacher A: “And what’s that tall thing over there?” C5: “It’s a three-layer cake for the party!”	Subtheme A-2: Activating descriptive vocabulary via successive, targeted prompts. Subtheme A-1: Extending discourse topics by directing attention to specific image elements.

Notes: C5 used a total of 1 metaphor, 2 adjectives, and 1 ordinal number during the scaffolding phase, indicating that the scaffolding pointing question significantly contributed to lexical diversity.

**Table 7:** C4 and C5 vocabulary change tracking sheet.

Child	Phase	Noun	Metaphors	Adjectives	Ordinal Numbers	Emotion Words
C4	Warm-up	1 (Low)	0 (Low)	1 (Low)	0 (Low)	0 (Low)
	Scaffolding	2 (Med)	2 (Med)	2 (Med)	1 (Med)	1 (Med)
	Sharing	3 (High)	1 (Med)	2 (Med)	1 (Med)	2 (High)
C5	Warm-up	1 (Low)	0 (Low)	0 (Low)	0 (Low)	0 (Low)
	Scaffolding	2 (Med)	1 (Med)	2 (Med)	1 (Med)	1 (Med)
	Sharing	3 (High)	1 (Med)	2 (Med)	1 (Med)	3 (High)

Notes: The values in the table are the number of occurrences of each word class in the corresponding session; the rank in parentheses indicates its frequency level. (Low: 0 occurrences; Med: 1–2 occurrences; High: ≥3 occurrences).

**Table 8:** Dialogs and scaffolding in narrative co-construction (C3).

Time	Talking Episodes	Teacher Scaffolding
00:08:15	Teacher A: “What kind of net are you drawing? Why is it brown?” C3: “It’s a tennis net. The rabbit likes to play tennis under the brown net.”	Subtheme B-1: Prompts children to construct the context of an event by asking questions.
00:15:35	Teacher A: “Wow, there are three numbers over here. What is the rabbit doing standing on it?” C3: “It’s in number one, standing on the first frame. The audience is clapping for it.” Teacher A: “How did it win? What sport did it play?” C3: “It jumped the highest, and passed that rabbit. (points to another rabbit)”	Subtheme B-2: Prompts children to add to the development of events by asking about process and cause and effect.
00:18:45	Teacher A: “What are the little animals next to it doing? What are they saying?” C3: “They are watching the race and one is jumping high.” Teacher A: “And are they friends with anyone?” C3: “Yes, they come to the race too, but they are not number one.”	Subtheme B-2: Leads children to add emotional descriptions and character relationships to enrich the narrative context.

**Table 9:** Dialogs and scaffolding in narrative co-construction (C1).

Time	Talking Episodes	Teacher Scaffolding
00:08:15	Teacher A: “This rabbit, why is it standing in the area?” C1: “It’s going to a party and I’m going to paint a helicopter.”	Subtheme B-1: Encourages children to construct narrative starting points.
00:12:24	Teacher A: “What is this square for? Is it a house?” C1: “No, it’s a helicopter to land, it’s the entrance to the party!” Teacher A: “Wow, so what does it do when it goes in?” C1: “It’s going to dance and eat carrot cake.”	Subtheme B-2: Guides children to add to the “carry-over” plot by asking follow-up questions such as “what is it” and “what’s next?”
00:20:21	Teacher A: “Is this cave its house? Or is it someone else’s house?” C1: “Yes, it is the home of its friends, and they are going to go together.” Teacher A: “Is it happy? Look at its big smile.” C1: “Well, very happy. Because …… this is the first time it has ever gone to a party, in a helicopter.”	Subtheme B-2: Encourage children to include character emotions and social intentions.

**Table 10:** Narrative coherence scores of the six children.

Child	Subtheme B-1: Open-Ended Questioning (1–3)	Subtheme B-2: Narrative Extension Scaffolding (1–3)
C1	2	3
C2	2	3
C3	2	3
C4	2	2
C5	1	3
C6	1	2
Average	1.7	2.5

Note: Scoring criteria: 1 = low coherence; 2 = moderate coherence; 3 = highly coherent.

**Table 11:** Dialogs and scaffolding in emotional–narrative expression (C2).

Time	Talking Episodes	Teacher Scaffolding
00:21:00	Teacher A: “Would you like to tell everyone about this painting? This rabbit you drew is so happy.” C2: “It won the race.”	Subtheme C-1: Encouraging questions to create an atmosphere of self-confidence.
00:27:24	Teacher A: “What does it want to do when it wins?” C2: “It wants to go home and tell its mom and eat carrot cake to celebrate.”	Subtheme C-2: Empathic response to guide emotional detail.
00:28:11	Other children: “Wow…” C2: “I want to paint the cake, and I want to celebrate too.”	Subtheme C-2: Positive peer feedback reinforces emotional engagement.
	C2: “And fireworks, they are jumping.”	

**Table 12:** Emotional scaffolding conversations during the drawing process (C6).

Time	Talking Episodes	Teacher Scaffolding
00:13:40	Teacher A: “This gift box is so pretty!” C2: “A gift for a friend, she’ll love it when she sees it!”.	Subtheme C-1: Emotional compliments to enhance willingness to express.
00:16:25	Teacher A: “I wonder what she’ll do when she sees it?” C2: “She will jump up and down with joy to see the gift!”	Subtheme C-2: Empathic questioning to stimulate emotional and psychological portrayal of the character.

**Figure A1:**
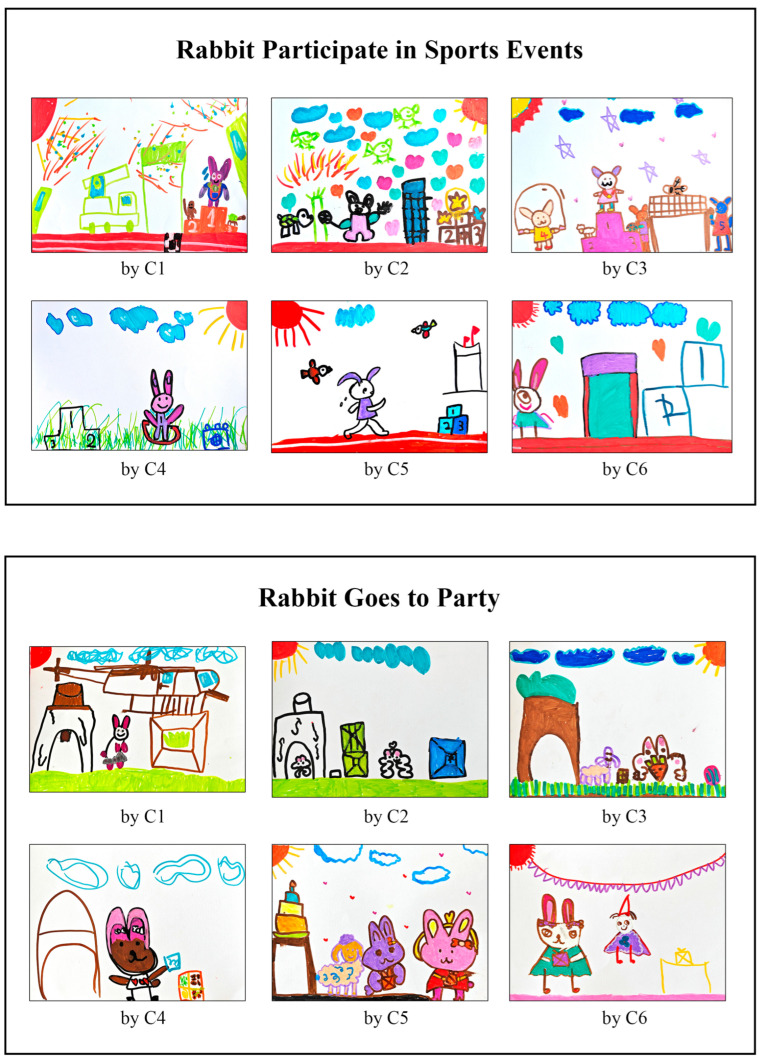
Children’s artworks (source: children’s drawings).

## Data Availability

The data supporting the findings of this study are available within the article.

## Abbreviations

The following abbreviations are used in this manuscript:

OLD	Oral Language Development
ZPD	Zone of Proximal Development
Chinese-CDI	Chinese version of the MacArthur–Bates Communicative Development Inventories
